# Investigating the differential impact of school and community-based integrated control programmes for soil-transmitted helminths in Timor-Leste: the (S)WASH-D for Worms pilot study protocol

**DOI:** 10.1186/s40814-016-0109-4

**Published:** 2016-12-08

**Authors:** Naomi E. Clarke, Archie C. A. Clements, Stuart Bryan, John McGown, Darren Gray, Susana V. Nery

**Affiliations:** 1 Research School of Population Health, Australian National University, Canberra, ACT 0200 Australia; 2 Cruz Vermelha Timor-Leste, Dili, Timor-Leste; 3 Plan International Timor-Leste, Dili, Timor-Leste

**Keywords:** Soil-transmitted helminths, Water, Sanitation and hygiene, Mass drug administration

## Abstract

**Background:**

Water, sanitation and hygiene (WASH) interventions represent an important component of soil-transmitted helminth (STH) infection control, alongside the administration of anthelmintic drugs, which are generally targeted to school-aged children. Recent modelling studies have suggested that STH control programmes should be broadened to include all age groups across the community. We describe the protocol for a pilot study investigating the impact of school-versus-community-based delivery of integrated WASH and deworming programmes on STH infections in school-aged children in Timor-Leste.

**Methods:**

The (S)WASH-D for Worms pilot is a two-arm, non-randomised cluster intervention study. The aims are to determine feasibility and acceptability of the intervention and study procedures and to establish proof of principle for the hypothesis that STH control programmes directed to the entire community will lead to greater reductions in STH infections in children than programmes directed only to school-aged children. Of the six participating communities, three receive a school-based integrated WASH and deworming programme and three additionally receive a community-based integrated WASH and deworming programme. The primary outcomes are the proportions of eligible children who enrol in the study and participate in the data collection, and outcomes relating to WASH and deworming programme completion, coverage, and use. Secondary outcomes are the cumulative incidence and mean intensity of STH infection in school-aged children at 6-month follow-up, mean haemoglobin concentration and several anthropometric indices. Results will inform the design of a cluster-randomised controlled trial (RCT).

**Discussion:**

This pilot study is being conducted in preparation for a cluster-RCT investigating the differential impact of school- and community-based integrated STH control programmes on STH infections in school-aged children. It aims to establish feasibility and proof of principle, while results of the subsequent RCT could have significant implications for global STH control policy.

**Trial registration:**

Australian New Zealand Clinical Trials Registry, ACTRN12615001012561

**Electronic supplementary material:**

The online version of this article (doi:10.1186/s40814-016-0109-4) contains supplementary material, which is available to authorized users.

## Background

Soil-transmitted helminths (STHs) represent a group of parasitic nematode worms which fall into the category of neglected tropical diseases—a group of infections which predominantly affect people living in extreme poverty [1]. The soil-transmitted helminths include hookworms (*Necator americanus* and *Ancylostoma duodenale*), roundworms (*Ascaris lumbricoides*) and whipworms (*Trichuris trichiura*). Together, it is estimated that approximately 1.45 billion people worldwide are infected with at least one of these species of worms [2], with an estimated disease burden in excess of five million disability-adjusted life years (DALYs) [3].

STH infections are transmitted when helminth eggs are excreted in the faeces of infected individuals, contaminating soil in areas without adequate sanitation. Infections are subsequently acquired through direct penetration of the skin by hookworm larvae, or accidental ingestion of *A. lumbricoides* or *T. trichiura* eggs, or hookworm larvae [4]. These infectious stages of STH can remain viable in soil for a period of time ranging from several weeks for hookworm larvae to several years for *A. lumbricoides* eggs [5–7].

Chronic STH infections result in malabsorption of nutrients and micronutrients, and a number of studies show evidence for malnutrition, iron-deficiency anaemia, poor growth and impaired cognitive development in STH-infected individuals [8–14], with children harbouring the largest burden of morbidity [4, 7, 15, 16]. Both *A. lumbricoides* and *T. trichiura* infections have a peak incidence and intensity in children between the ages of 5 and 15, with a decline in both frequency and intensity in adulthood [4]. Hookworm infections, on the other hand, tend to maintain a high prevalence and intensity in adulthood [4, 5]. Despite this, children, along with women of child-bearing age, remain at the highest risk of hookworm-associated anaemia and other related morbidities [5, 17].

Regular treatment with the benzimidazole anthelmintic albendazole leads to rapid and significant decreases in STH prevalence, particularly *A. lumbricoides* and hookworm [18]. Regular anthelmintic delivery—also known as deworming—has been shown in a number of studies to improve STH morbidity indicators, including growth, anaemia, cognitive abilities and school attendance [11, 15, 19, 20], although some of this evidence has recently been called into question [21–23].

Following the administration of anthelmintic drugs, STH infections rapidly recur in the context of ongoing environmental contamination [24]. Therefore, to achieve sustainable control of STH infections, facilitating improvements in water, sanitation and hygiene (WASH) in order to interrupt the cycle of ongoing reinfection is thought to be important [25, 26]. The impact of adequate water and sanitation infrastructure, as well as good hygiene practice, on preventing enteric infections and diarrhoeal illness is widely understood [27–29]. Intervention studies and systematic reviews specifically examining the link between WASH components and STH infections show evidence, albeit not consistent, that access to improved water and sanitation, and exposure to health education or hygiene promotion, are associated with reduced odds of STH infection [30, 31] or reduced risk of reinfection following drug treatment [32–36].

The optimal strategy for delivery of integrated deworming and WASH approaches remains uncertain. Due to the heavy burden of STH morbidity in school-aged children, and the cost-effectiveness of using school-based infrastructure [37], the World Health Organization (WHO) guidelines have focused predominantly on school-aged children as major targets of anthelmintic drug programmes [14, 38], with more recent recommendations including preschool-aged children, women of child-bearing age and adults in high-risk occupations [16]. School-based deworming programmes have been widely advocated and have become a cornerstone of STH control [15, 16]. The London Declaration on Neglected Tropical Diseases (NTDs) in 2012 saw 600 million annual doses of anthelmintic drugs donated towards the control of STH in children [39], a step towards achieving the WHO target of 75% deworming coverage of at-risk preschool- and school-aged children by 2020 [16]. This has resulted in a large global scale-up of chemotherapy programmes targeting school- and preschool-aged children [40].

However, recent modelling studies have raised concerns about the impact of child-targeted control programmes on the transmission of STH in the wider community [41–44]. These studies suggest that targeted programmes may not significantly impact the overall level of transmission [41, 42] and that child-focused strategies may be ineffective in reducing the overall community burden of the disease, particularly in areas where hookworm infections are predominant [43, 44]. Therefore, expanding treatment programmes to the whole community may result in improved STH control [42]. Cost-effectiveness modelling has demonstrated that community-based drug administration programmes for STH control are highly cost-effective when compared with treatment of school-aged children only [44, 45].

Intervention studies examining the impact of one or more components of WASH on STH infections have been conducted, or are currently underway, both in schools [32–36] and in communities [46–49]; however, the relative merits of the two delivery strategies have not been discussed in the literature. Furthermore, to our knowledge, there are no studies which have directly compared school-based and community-based integrated WASH and deworming programmes. The (School) Water, Sanitation, Hygiene and Deworming for Worms ((S)WASH-D for Worms) study aims to contribute to this evidence gap by comparing an integrated approach focused on school children with an integrated community-based approach. This report describes the protocol of the (S)WASH-D for Worms pilot study, which is being conducted in preparation of a full-scale cluster-randomised controlled trial. This protocol has been developed using the SPIRIT 2013 guidelines (see Additional file 1) [50].

The objectives of this pilot study are as follows:

1. To examine the feasibility and acceptability of conducting a trial that recruits school-aged children and implements distribution of deworming medications along with school- and community-based WASH programmes. Specifically,
  - To determine the feasibility and acceptability of study procedures by estimating rates of participant consent, recruitment, participation in data collection and retention
  - To determine the feasibility and acceptability of the WASH and deworming programme by observing completion, uptake and usage
  - To identify operational issues for consideration when planning the full-scale trial
  - To obtain the initial estimates of STH prevalence, infection intensity and nutritional indicators for the purpose of informing sample size calculation
2. To establish “proof of principle” (preliminary evidence) for our hypothesis that a community-based deworming and WASH intervention is more effective in reducing STH infections in children than an exclusively school-based approach, by comparing estimates of the impact of the interventions

## Methods

### Design

**Fig. 1:**
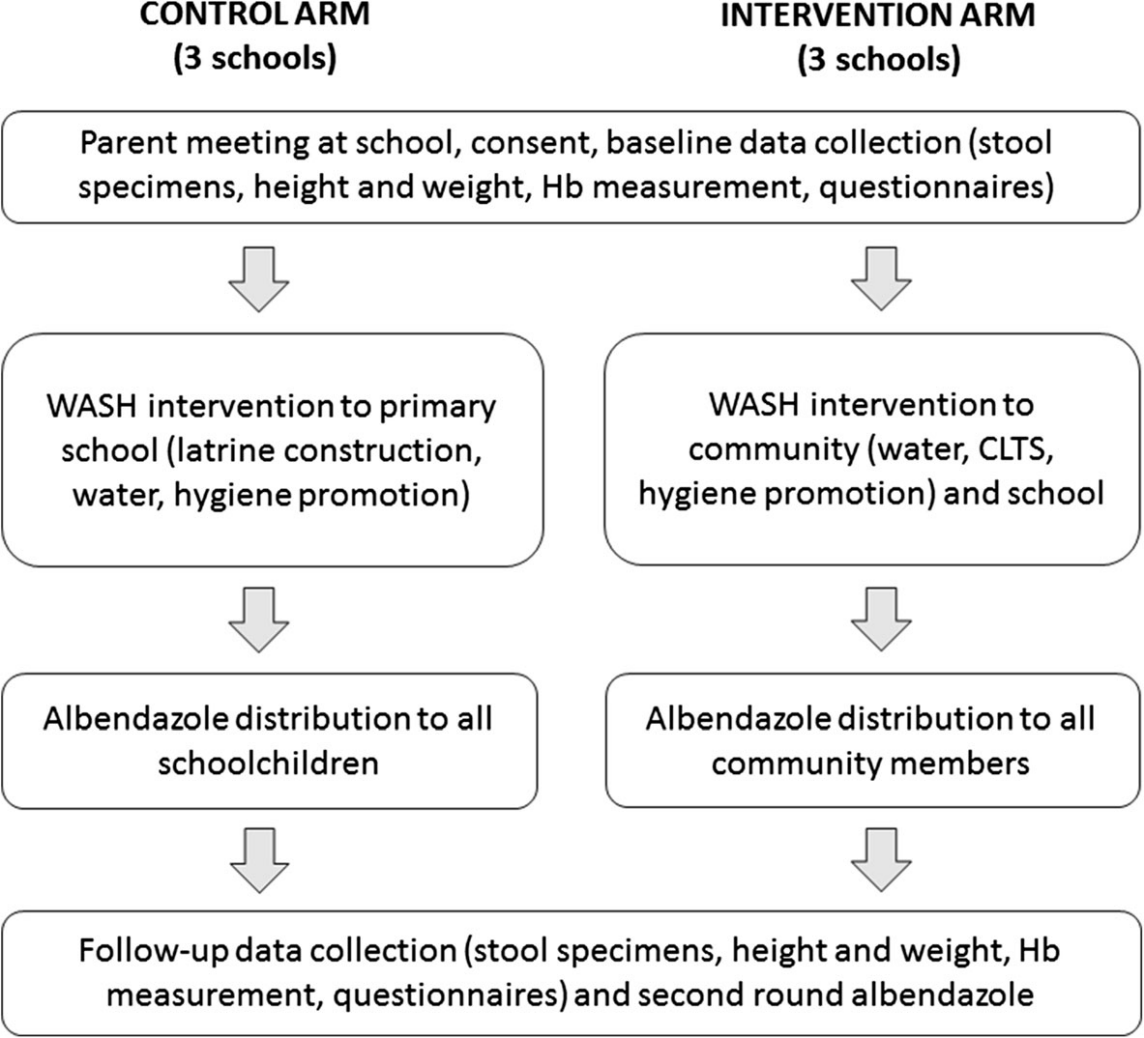
Flow diagram of the pilot study

This pilot study is a two-arm, non-randomised cluster intervention study. The six participating clusters, each based around a local primary school, are located in Aileu and Manufahi municipalities of Timor-Leste. Three clusters comprise the “control” arm of the study: in these, a WASH programme is delivered to the primary school, and albendazole is distributed to the schoolchildren. The other three clusters comprise the “intervention” arm, in which a WASH programme is delivered to both the primary school and the community in which the school is located, and albendazole is distributed to all community members. The follow-up period for the pilot study is 6 months following the distribution of albendazole, which will allow sufficient time for STH reinfection to occur [24], and represents the follow-up interval planned for the full-scale trial, which will take place over a 2-year period. Figure 1 depicts a flow diagram for the pilot study. This pilot study is registered with the Australian New Zealand Clinical Trials Registry (registration number ACTRN12615001012561).

### Setting

Timor-Leste is placed 133rd out of 187 countries on the Human Development Index [51], with 31.5% of the population living in severe poverty and a further 21.4% living in near poverty [51]. Over 50% of children under 5 years of age suffer from stunting, indicating chronic malnutrition [52]. A national survey in 2012 showed an overall STH prevalence of 29% in school-aged children in Timor-Leste, with 26 and 30% prevalence in the Manufahi and Aileu municipalities, respectively [53].

Open defecation in rural communities poses significant health risks. In 2015, 73% of the rural households in Timor-Leste did not have access to improved sanitation facilities, and 39% did not have access to improved water supplies [54]. Furthermore, a UNICEF survey in 2011 reported that 35% of the primary schools in Timor-Leste did not have latrines, and 62% of schools did not have regular access to a water supply [55].

Improved water and sanitation facilities across rural areas of Timor-Leste has been highlighted as a priority by the government of Timor-Leste [56], and multiple non-governmental organisations (NGOs) are also working in Timor-Leste to provide rural communities with improved access to reliable protected water sources and sanitation facilities and to promote hygiene behaviour [57–61].

### Integrated STH control programme

The WASH programmes in the pilot study are implemented by partner NGOs working in Timor-Leste. In order to ensure that the WASH programme would be completed within the planned time frames, two partner NGOs were selected. Plan International Timor-Leste is the implementer of the school- and community-based WASH programmes in the intervention arm of the study. These programmes are being delivered as part of a 4-year EU-funded water and sanitation project (FED/2011/270-630). Cruz Vermelha Timor-Leste (CVTL; a partner of Red Cross Australia) is the implementer of school-based WASH programmes in the control arm of the study, which are being delivered as a special project within the Integrated Community Based Risk Reduction program, funded by the Australian NGO Cooperation Program (ANCP 777-PRG01-PRJ08).

#### School WASH programme

All study clusters receive a school-based WASH programme, which includes three of the following components:

(A) Providing access to a protected source of water which will be available year-round. This involves the construction of a new water system where required or the development or rehabilitation of existing water systems to improve water supply to the schools
(B) Providing access to sanitation, achieved through either the construction of new school latrines or rehabilitation of existing, non-functional school latrines. Latrines are built following the Timor-Leste WASH in schools guidelines, which provide standards for the construction of sufficient, gender-segregated, accessible, private, secure, clean and culturally appropriate toilets for schoolchildren and staff, including facilities for use by menstruating students and staff [62]. Toilets are designed so that they are hygienic to use and easy to clean. At study schools, pour-flush latrines are constructed (or rehabilitated). The latrine pits are lined with concrete rings, and the superstructures are built with concrete blocks for durability. Handwashing stations are also constructed
(C) Improving hygiene behaviour through hygiene promotion sessions conducted at primary schools. These sessions focus on using latrines, handwashing with soap at key times and keeping the environment clean. Strategies to communicate these messages include the use of flip charts, banners and posters, as well as game-oriented activities and participatory demonstrations

#### Community WASH programme

In addition to the school-based WASH programme, the three intervention clusters also receive a WASH programme delivered at the community level, including the same three components:

(A) Providing access to a protected primary source of water that will be available year-round. This involves the construction of a new water system, or rehabilitation of an existing one, and comprehensive training for community-based water committees about operation and maintenance
(B) Access to improved sanitation, achieved by increasing the number of household latrines. Plan International Timor-Leste and its partners utilise the community-led total sanitation (CLTS) approach, which encourages all households to take responsibility for building and using their own household latrines, thus eliminating open defecation in their communities [63]. CLTS challenges community members to reflect on their defecation practices through a series of sessions collectively called “triggering” that include transect walks, mapping open defecation areas, calculating the amount of faeces produced daily by each household, explanations on faecal-oral disease transmission routes and costs of medical treatment for gastrointestinal illnesses. If triggering is conducted optimally, community members come to the realisation that they are consuming each other’s faeces. Finally, community members make an activity plan and pledge to build or repair their household toilets [63]. CLTS facilitators provide information and lead discussions around the types of latrines which could be built and local materials that could be used in their construction. The two most common types of latrines that are built are simple direct pit latrines and offset pit pour-flush latrines
(C) Hygiene promotion, conducted at the community level by Plan International and its partner NGOs as well as village health volunteers. This involves regular house-to-house visits to promote construction and use of latrines, handwashing with soap at key times and treatment and storage of drinking water

#### Administration of albendazole

In control clusters, albendazole is distributed to school-aged children only, while in intervention clusters, albendazole is distributed to every eligible member of the community. This includes all community residents, except for children under 1 year of age and pregnant women in the first trimester, in line with the WHO guidelines [64]. All doses are given as a single oral dose of 400 mg albendazole (Albenza, GlaxoSmithKline, Research Triangle Park, NC), taken under direct observation of the field staff.

In control clusters, albendazole distribution occurs at the primary school on a day agreed with the head teacher, with the first round given following the completion of school latrines and water systems. In intervention clusters, albendazole distribution occurs both at the primary school, again on a day agreed with the head teacher, and house-by-house, over a period of 1 to 2 days, with the first round given once 80% of households have latrines, and following the completion of school latrines and water systems. In both arms of the study, a second round of albendazole will be given at follow-up 6 months later, following the collection of follow-up stool specimens from the study participants.

Albendazole is widely distributed in large mass drug administration programmes globally; side effects are minor [64]. Parents are advised to seek healthcare at the local community health centre if their child is unwell following drug distribution, and community health centres are notified of study activities during field visits.

### Study outcomes

The primary outcomes will be used to examine feasibility and acceptability (objective 1). Primary outcomes relating to study feasibility and acceptability in terms of recruitment and participation (objective 1A) are as follows: the proportion of eligible children whose parents provide informed consent; the proportion of eligible children who provide stool samples, complete questionnaires and undergo measurement of height, weight and haemoglobin; and the retention rate of participants between baseline and follow-up.

Primary outcomes relating to the feasibility and acceptability of the WASH and deworming programmes (objective 1B) are as follows: the proportion of children and eligible community members who receive albendazole; the time taken for the completion of the school WASH programmes in each study cluster; the time taken to achieve 80% household latrine coverage in each intervention cluster; the proportion of schools and households with latrines and access to a reliable source of improved water; the proportion of schools with handwashing stations; and the proportion of children who report using latrines and handwashing stations.

Secondary outcomes, which will be used to inform sample size calculation (objective 1D) and to examine the study hypothesis (objective 2), are as follows: 6-month cumulative incidence of infection with STH (*Ascaris* spp., *N. americanus*, *Ancylostoma* spp. and *T. trichiura*) at follow-up; mean intensity of STH infection (measured as average number of eggs per gramme of faeces) at follow-up; mean haemoglobin concentration; and four anthropometric indices: weight-for-age, height-for-age, weight-for-height and body mass index (BMI)-for-age *Z*-scores (to identify underweight, stunting, wasting and thinness, respectively). Cumulative incidence of STH infection is the planned primary outcome in the full-scale trial. It should be noted that the term “cumulative incidence” is used for simplicity, as cases diagnosed at follow-up will include both incident infections and prevalent infections not cured by albendazole, particularly in the case of hookworm and *T. trichiura* infections [65].

All outcomes (see Table 1) will be compared between control and intervention clusters.

**Table 1:** Study outcomes

Primary outcomes	
Proportion of eligible children who:	Provide parental informed consent
	Provide stool samples
	Complete questionnaires
	Undergo measurement of height, weight and haemoglobin
Participant retention rates between baseline and follow-up (defined as the proportion of baseline participants who were retained in the study at follow-up)	
Proportion of children and eligible community members who take albendazole	
Time taken to complete school WASH programmes in each study cluster	
Time taken to achieve 80% household latrine coverage in each intervention cluster	
Proportion of schools and households with functional and clean latrines	
Proportion of children who report using household and school latrines	
Proportion of schools and households with access to a reliable primary source of improved water	
Proportion of schools with handwashing stations	
Proportion of children who report using handwashing stations at school	
Secondary outcomes	
Cumulative incidence of infection with:	*Ascaris* spp.
	*T. trichiura*
	*N. americanus*
	*Ancylostoma* spp.
Mean intensity of infection (calculated as the average number of eggs per gramme of faeces) of:	*Ascaris* spp.
	*T. trichiura*
	*N. americanus*
	*Ancylostoma* spp.
Mean haemoglobin concentration	
Weight-for-age (underweight) *Z*-score	
Weight-for-height *Z*-score (wasting)	
Height-for-age *Z*-score (stunting)	
Body mass index (BMI)-for-age *Z*-score (thinness)	

### Selection and recruitment of clusters

Because each of the two partner NGOs only had capacity to conduct the WASH programme in one of the study arms within the required study time frame, and operated in neighbouring but different administrative areas, a randomised design could not be used for the pilot project. In the full-scale study, the intention is to randomise communities to the intervention and control arms.

For the pilot study, communities were considered eligible if they contained a primary school which was suitable for a school-based WASH programme (i.e. did not have access to functional improved latrines) and were located in a village with low sanitation coverage (i.e. less than 50% of households with latrines). Communities were selected in consultation with the implementing partner NGOs, based on their upcoming activities which fit into the study time frame.

Members of the (S)WASH-D for Worms research team accompanied NGO staff to community meetings in each cluster. For intervention communities, this occurred at the community “triggering”, and for control communities, this was a pre-arranged meeting with community and school leaders to explain plans for the school-based WASH programme. In all clusters, the study was explained to the village leader and head of school, who provided consent for the study activities to take place in their community. During the triggering in intervention communities, trial staff were also given the opportunity to explain the research study to the community. Following these initial meetings, plans were made for the research team to return for baseline data collection at the schools, within 1 to 3 weeks of the first meeting and prior to the commencement of the WASH programme.

### Participants

Participants in the data collection in both arms of the study are children attending the local primary school. The study has no specific exclusion criteria; all children enrolled in the local primary school are eligible for participation, provided a parent or guardian is available to provide informed consent.

Prior to the baseline field visit, teachers were asked to organise a parent meeting at the school on the day the research team arrived. At this meeting, the study was explained in detail to the parents by the Timorese project manager. Parents were provided with both written and schematic information sheets and given the opportunity to ask questions about the study prior to providing written informed consent.

At baseline, informed consent was obtained for 522 out of 602 eligible children (i.e. those who were enrolled in the local primary school), representing a recruitment rate of 87%. Of the 80 children who were not recruited to the study, 39 were absent from school during the baseline visit, and 41 were present but their parents were unable to attend the school to provide informed consent. No refusals of consent were recorded among children whose parents attended.

At the 6-month follow-up visit, which will take place in a new academic year, teachers will again be asked to arrange a parent meeting, and consent will be sought from any parents who did not attend at baseline, as well as from parents of children who are new to the school, including all children in the new grade 1 cohort.

### Data collection

#### Questionnaires

Study participant questionnaires are administered as interviews at both baseline and follow-up. They are conducted by trained local fieldworkers and include two components. The first component consists of questions asked directly to the children, relating to diarrhoea history, access to deworming medications, presence and use of a household latrine, defecation practices, handwashing practices and shoe wearing at home, at school and while defecating. The second component consists of questions directed to the caregiver, relating to household water source, household assets, education and occupation. Questionnaires are also administered to school and village leaders and include questions relating to school and community latrines and water sources.

#### Stool samples

Stool samples are collected at both baseline and 6-month follow-up. On the first day of each field visit, all participating children are given an explanation of the study and requested to provide a stool sample as part of their participation. Each child is given a labelled plastic container and provided with instructions on the collection of a faecal sample, ideally to be done the following morning and returned to the field team at the school.

Upon receipt of the stool specimens by the field team, two aliquots of 2–3 g are taken and preserved in 15-mL centrifuge tubes—one containing 8 mL of 10% formalin, and the other containing 5 mL of 5% potassium dichromate.

The formalin-fixed samples are transported to the University of Melbourne, Victoria, Australia, for diagnostic processing using microscopy. This is achieved using a simple sodium nitrate flotation technique and direct microscopy to quantify the number of STH eggs (*A. lumbricoides*, hookworm spp. and *T. trichiura*) in each faecal sample [66].

The potassium dichromate-fixed samples are sent to the QIMR Berghofer Medical Research Institute, Brisbane, Australia, for diagnostic processing using a polymerase chain reaction (PCR) technique. DNA is extracted using the PowerSoil DNA extraction kit, with modifications [67], and a real-time multiplex PCR is then undertaken to detect and quantify soil-transmitted helminths (*Ascaris* spp., *N. americanus*, *Ancylostoma* spp. and *T. trichiura*) [68].

#### Measurement of height, weight and haemoglobin

At both baseline and follow-up field visits, all children for whom informed consent has been provided undergo measurement of height (to the nearest 0.1 cm) and weight (to the nearest 0.5 kg), obtained as a single measurement. A fingerprick blood sample is also obtained for measurement of haemoglobin. These measurements are done by the (S)WASH-D for Worms field team, which includes a nurse, utilising a portable height rod (Wedderburn, WSHRP), digital scale (Livingstone, SCLBATHDIG) and a portable haemoglobin analyser (Hb 201+, HemoCue, Angelholm, Sweden).

Height and weight measurements will be used, along with age, to calculate anthropometric values indicative of nutritional status in children: weight-for-age, height-for-age, weight-for-height and BMI-for-height. These will be calculated as *Z*-scores, the number of standard deviations from the mean of the standard population, with malnutrition and severe malnutrition defined as values 2 and 3 standard deviations, respectively, below the mean score of the standard population [69], using the 2006 WHO database for child growth standards [70]. Anaemia is defined as per the WHO classification guidelines, adjusted for altitude in communities more than 1000 m above sea level.

### Data management and confidentiality

Questionnaire data, as well as height, weight and haemoglobin measurements and results of parasitological examinations, are entered into a password-protected database. Data are entered twice by two different data clerks, and the database has in-built range checks for appropriate variables. The final study dataset will be accessible only by the study investigators. Original questionnaires are kept in a locked cabinet in the study office in Timor-Leste and will be destroyed after 7 years. Stool samples are labelled using the participant’s unique study ID number, with no identifying information. Results of the parasitological examinations are entered into the study database described above.

### Analysis

For the primary outcomes, analyses will be mainly descriptive. The proportions of eligible participants who gave informed consent and participated in each aspect of data collection will be calculated (with 95% confidence intervals (CIs)) and compared across the two study arms, at both baseline and follow-up, and will also be examined separately by gender and age group. The proportion of baseline participants retained at follow-up will also be calculated and compared across the study arms. Descriptive statistics will be used to examine the completeness of data collected using the study questionnaires.

The proportion of children and eligible community members who received albendazole, the proportion of schools and households with access to various WASH components and the proportion of children who report using various WASH components will be calculated (with 95% CIs) and compared across the two study arms, at both baseline and follow-up. The time taken to complete the school and community WASH programmes will be examined using descriptive statistics (mean, median, range).

For the secondary outcomes, prevalence at baseline and cumulative incidence at follow-up will be calculated (with 95% CIs) for each STH as will the mean and standard deviation of the infection intensity, expressed as eggs per gramme of faeces. Mean and standard deviation of the haemoglobin concentration and *Z*-scores for the four anthropometric indices will also be calculated. These outcomes will be compared across both arms of the trial using mixed effects multivariable regression models that account for clustering of participants within schools and villages. Cumulative incidence of infection will be modelled using multivariable Bernoulli logistic regression, with age and sex entered as covariates, baseline infection status as a fixed effect, and school and village as random effects. The study arm will be entered as a binary fixed effect to estimate differences in cumulative incidence between the study arms, using a cumulative incidence ratio (CIR). Intensity of infection will also be modelled with random and fixed effects as described above. Mixed effects linear regression will be used to model anthropometric *Z*-scores and mean haemoglobin concentration. Stata software will be used for all analyses (StataCorp LP, College Station, TX).

### Dissemination

The results of this pilot study will be published in peer-reviewed journals and presented at national and international conferences. Results will also be conveyed to, and discussed with, the Timor-Leste Ministry of Health and relevant WASH programme stakeholders.

## Discussion

The current WHO guidelines for STH control focuses strongly on school- and preschool-aged children, who experience the highest burden of disease-related morbidity. In the context of significant global interest in the control of neglected tropical diseases, including significant donations from pharmaceutical companies, and recognition of the potential added benefit of WASH interventions for sustainable control, there is increasing interest in the optimal control strategies for STH.

The (S)WASH-D for Worms pilot study primarily represents a feasibility study in preparation for a cluster-randomised controlled trial (RCT) investigating the differential impact of school- and community-based integrated STH control programmes. The integrated control programme implemented in the study includes both deworming medications, distributed by the research team, and a water, sanitation and hygiene intervention, implemented by partner NGOs. The pilot study will provide an indication of the rate of recruitment and participation which could be expected in a full-scale RCT, which will be used to inform sample size calculations for the full-scale trial. The pilot study will also provide an opportunity to test the study procedures and data collection forms and to examine the feasibility and acceptability of the deworming and WASH programmes, in particular, the time frames for completion of the WASH programmes and their ability to achieve improved WASH access and use. Furthermore, it will allow for the identification of operational challenges involved in implementing such a trial in a developing country. In particular, the pilot study will give an estimation of the time frames required for the completion of the school- and community-based WASH interventions.

The pilot study sample size does not allow sufficient power to detect significant differences in secondary outcomes between study arms. Initial estimates of the secondary outcomes obtained in this pilot study will be used to provide preliminary evidence for our study hypothesis that a community-wide intervention is more effective at reducing STH infections in children than a school-based intervention and to inform sample size calculation. Results of hypothesis testing will be interpreted with caution; emphasis will be given to confidence intervals, rather than *p* values, and results will be presented in terms of assessment of “proof of principle”, rather than establishment of causation.

## Conclusion

Expanding existing school-based STH control programmes to all community members has the potential to result in improved STH control among school-aged children. The (S)WASH-D for Worms pilot study is the precursor to a cluster-RCT which will contribute to the current evidence gap and could have significant implications for global STH policy.

## Additional file

Additional file 1: Table S1. SPIRIT 2013 Checklist—recommended items to address in a clinical trial protocol and related documents*. (DOC 123 kb)

## Abbreviations

BMI: Body mass index
CI: Confidence interval
CIR: Cumulative incidence ratio
CLTS: Community-led total sanitation
CVTL: Cruz Vermelha Timor-Leste
DALY: Disability-adjusted life years
NGO: Non-governmental organisation
NTDs: Neglected tropical diseases
PCR: Polymerase chain reaction
RCT: Randomised controlled trial
STH: Soil-transmitted helminth
WASH: Water, sanitation and hygiene
WHO: World Health Organization
